# Supplementary material for: A clinically useful and biologically informative genomic classifier for papillary thyroid cancer
Source: Front Endocrinol (Lausanne). 2023 Sep 12;14:1220617. doi: 10.3389/fendo.2023.1220617 (PMC10523308; doi:10.3389/fendo.2023.1220617)
Supplement: Supplementary file 1 [file DataSheet_1.pdf]

Supplementary Material

A Clinically Useful and Biologically Informative Genomic Classifier for Papillary Thyroid Cancer

Steven Craig[1,2]†; Cynthia Stretch[3]†; Farshad Farshidfar[3]; Dropen Sheka[4]; Nikolay Alabi[4]; Ashar Siddiqui[5]; Karen Kopciuk[3,7]; Young Joo Park[8,9]; Moosa Khalil[10]; Faisal Khan[10,11]; Adrian Harvey[6], Oliver F. Bathe[3,6,12]

*** Correspondence:**
Corresponding Author
Oliver F. Bathe, Division of Surgical Oncology, Tom Baker Cancer Centre, 1331-29th St NW, Calgary, AB, Canada, T2N 4N2. Phone: 403-521-3275; Email: bathe@ucalgary.ca

Supplementary Figures and Tables

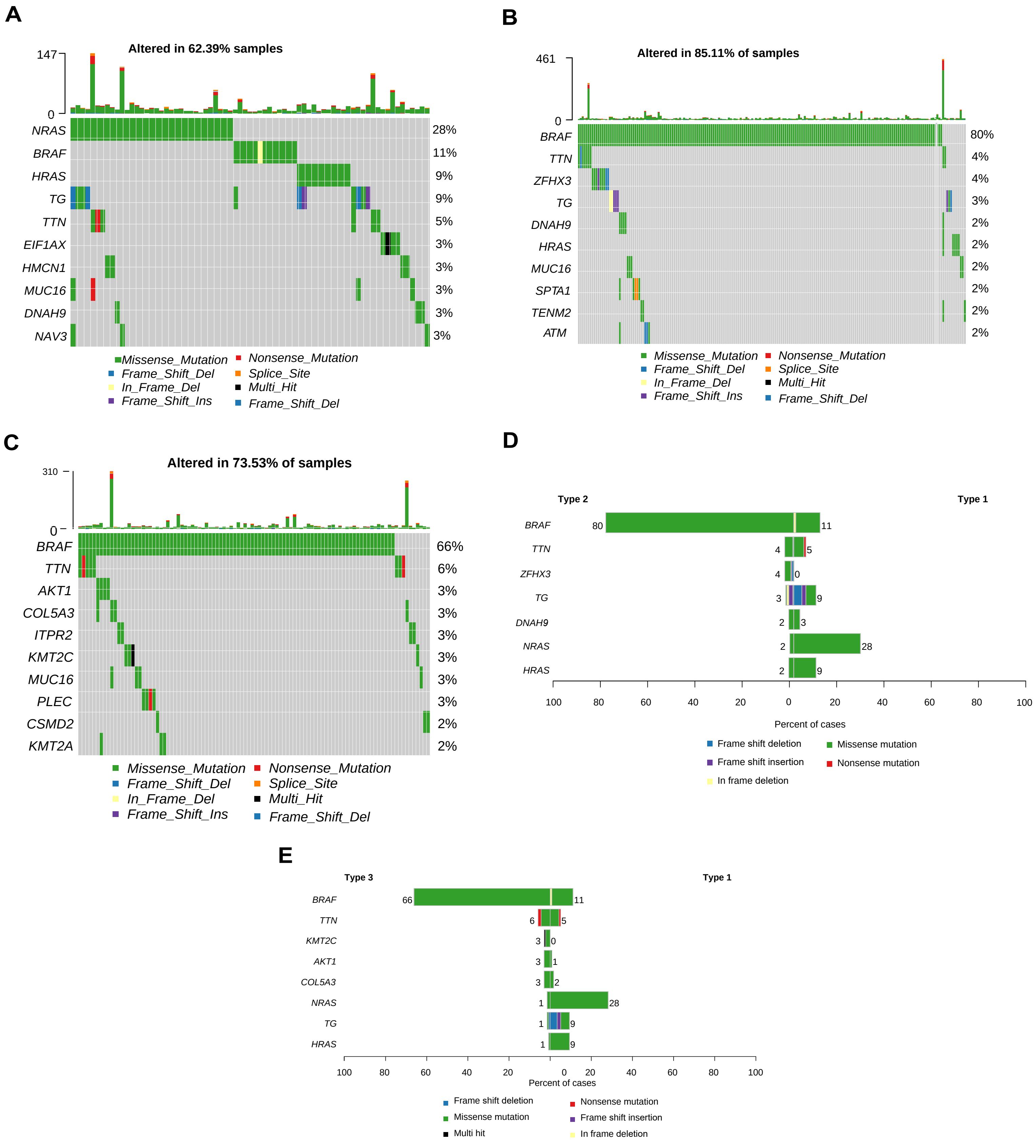

Supplementary Figure 1. Mutation analysis summary for the three molecular subtypes. **A** Oncoplot for Type 1 tumors. **B** Oncoplot for Type 2 tumors. **C** Oncoplot for Type 3 tumors. **D.** Co-oncoplot showing the genes with significantly different mutation rates between Type 2 tumors and Type 1 tumors. **E** Co-oncoplot showing the genes with significantly different mutation rates between Type 3 tumors and Type 1 tumors ($P < 0.05$).

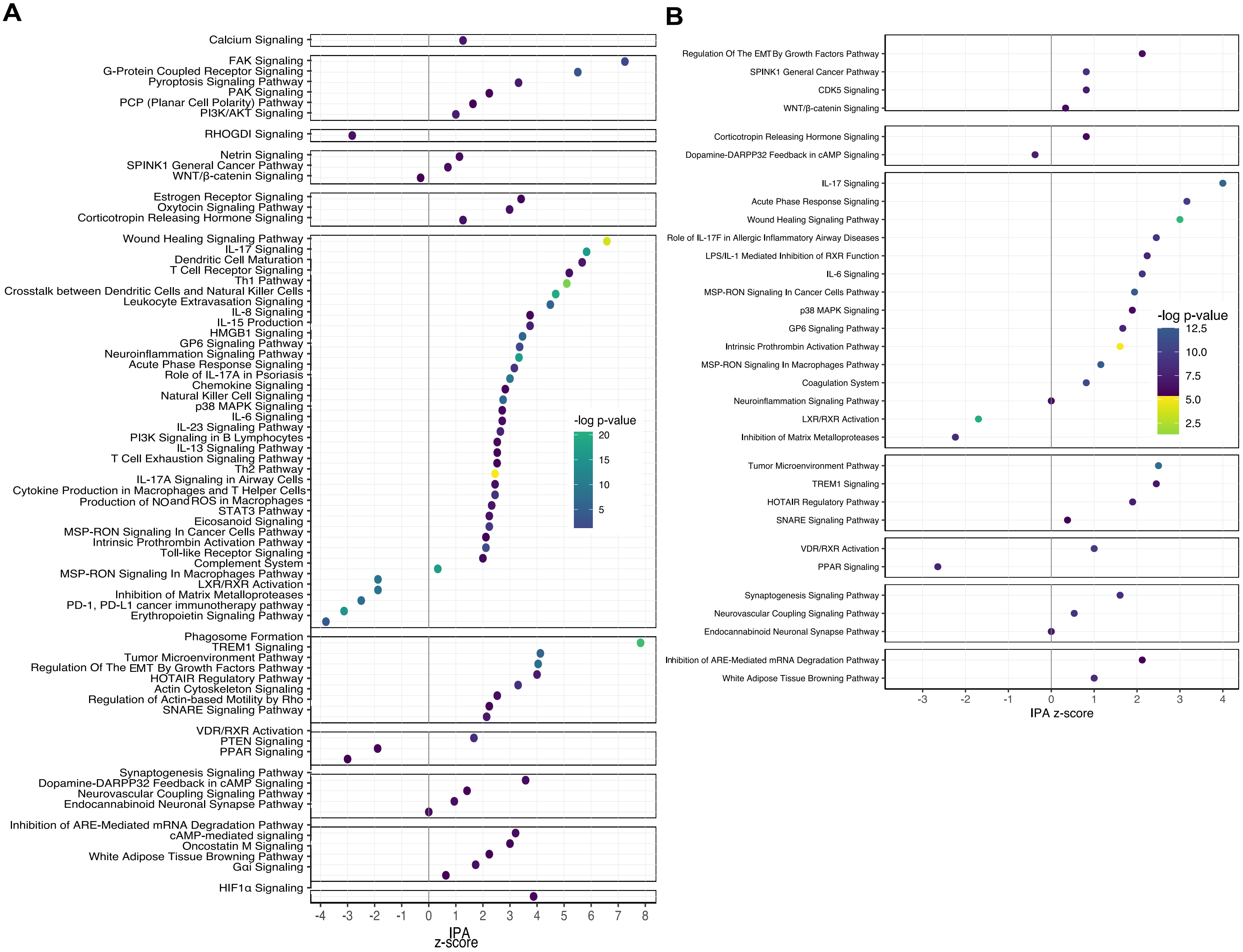

Supplementary Figure 2. Summary of pathway analysis results generated using Ingenuity Pathway Analysis (IPA) of differentially expressed mRNA between molecular subtypes. **A** Canonical pathways associated with differentially expressed genes (FDR <0.05, logFC >|2|) in Type 3 tumors versus Type 1 tumors. **B** Canonical pathways associated with differentially expressed genes (FDR <0.05, logFC >|2|) in Type 2 tumors versus Type 1 tumors. P-value was calculated by IPA using the Fisher's Exact Test.

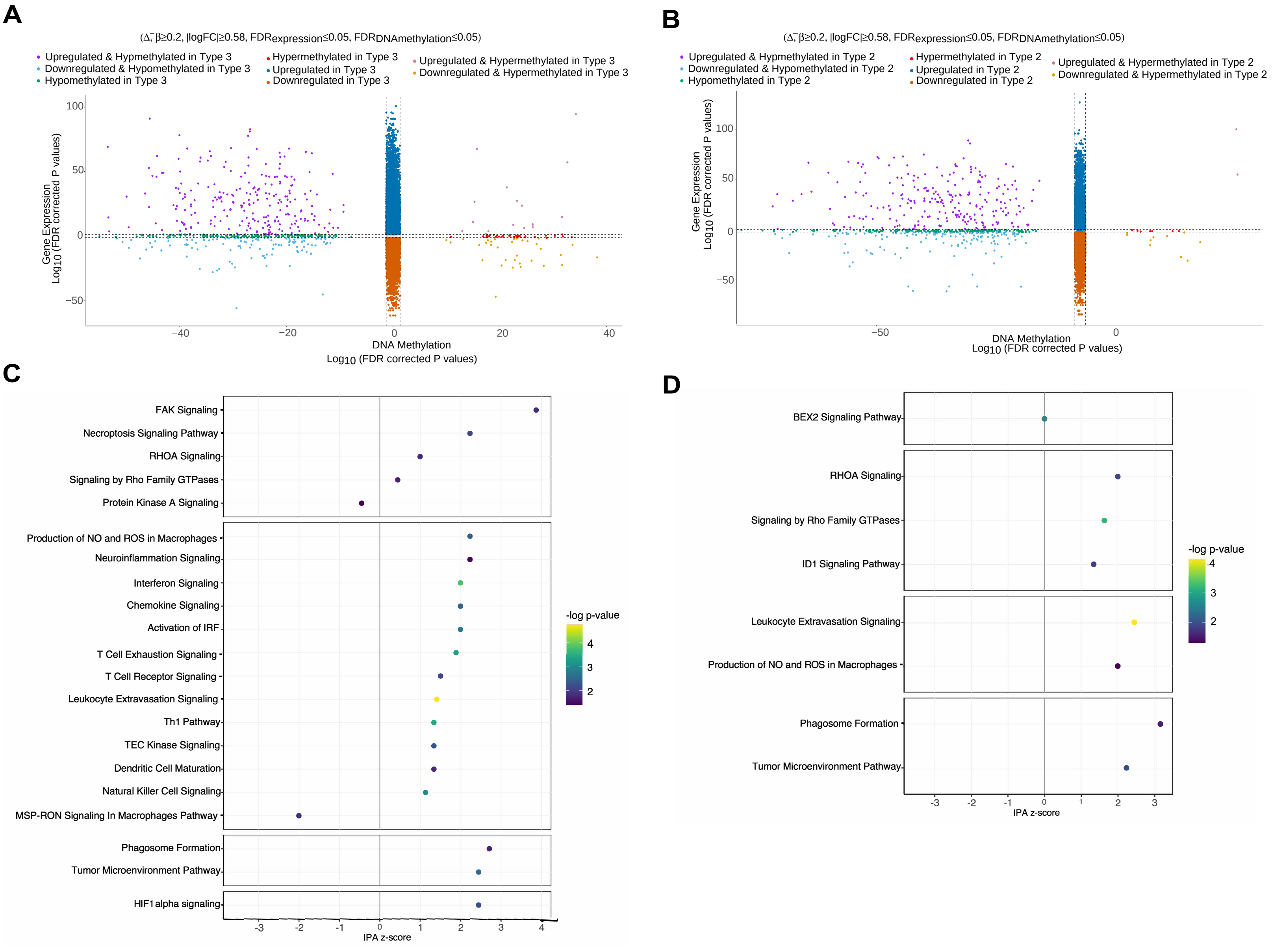

Supplementary Figure 3. Summary of methylation analysis of molecular subtypes. **A** Starburst plots for comparison between differential DNA methylation (x axis) and differential gene expression (y axis) when comparing Type 3 tumors with Type 1 tumors. **B** Starburst plots for comparison between differential DNA methylation (x axis) and differential gene expression (y axis) when comparing Type 2 tumors with Type 1 tumors. For **A** and **B**, the dashed black lines represent the cutoff of FDR adjusted p-values <0.05. **C.** Summary of pathway analysis results generated using Ingenuity Pathway Analysis (IPA) of differentially expressed mRNA targets of methylated genes identified by differential methylation analysis between Type 3 versus Type 1 tumors and **D** between Type 2 versus Type 1 tumors.

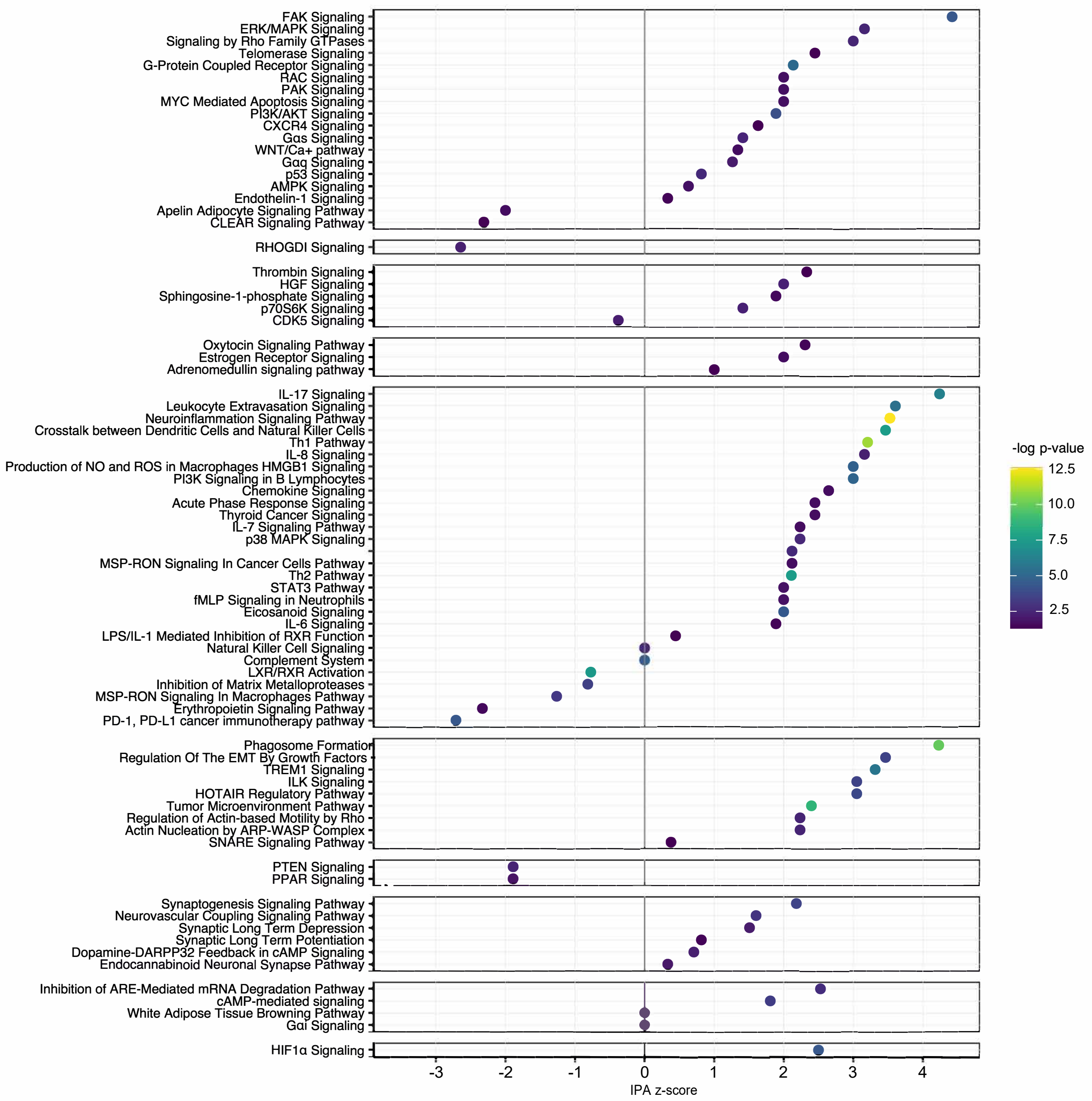

Supplementary Figure 4. Summary of differential miRNA analysis between Type 3 versus Type 1 tumors. Pathway analysis results generated using Ingenuity Pathway Analysis (IPA) of differentially expressed mRNA targets of miRNAs identified by differential miRNA analysis. Negative and positive z-scores (x axis) represent predicted decreased and increased activation of a canonical pathway, respectively. The -log p-value generated by IPA represents results of a Fisher's Exact Test analysis where $p<0.05$ indicates a statistically significant association.

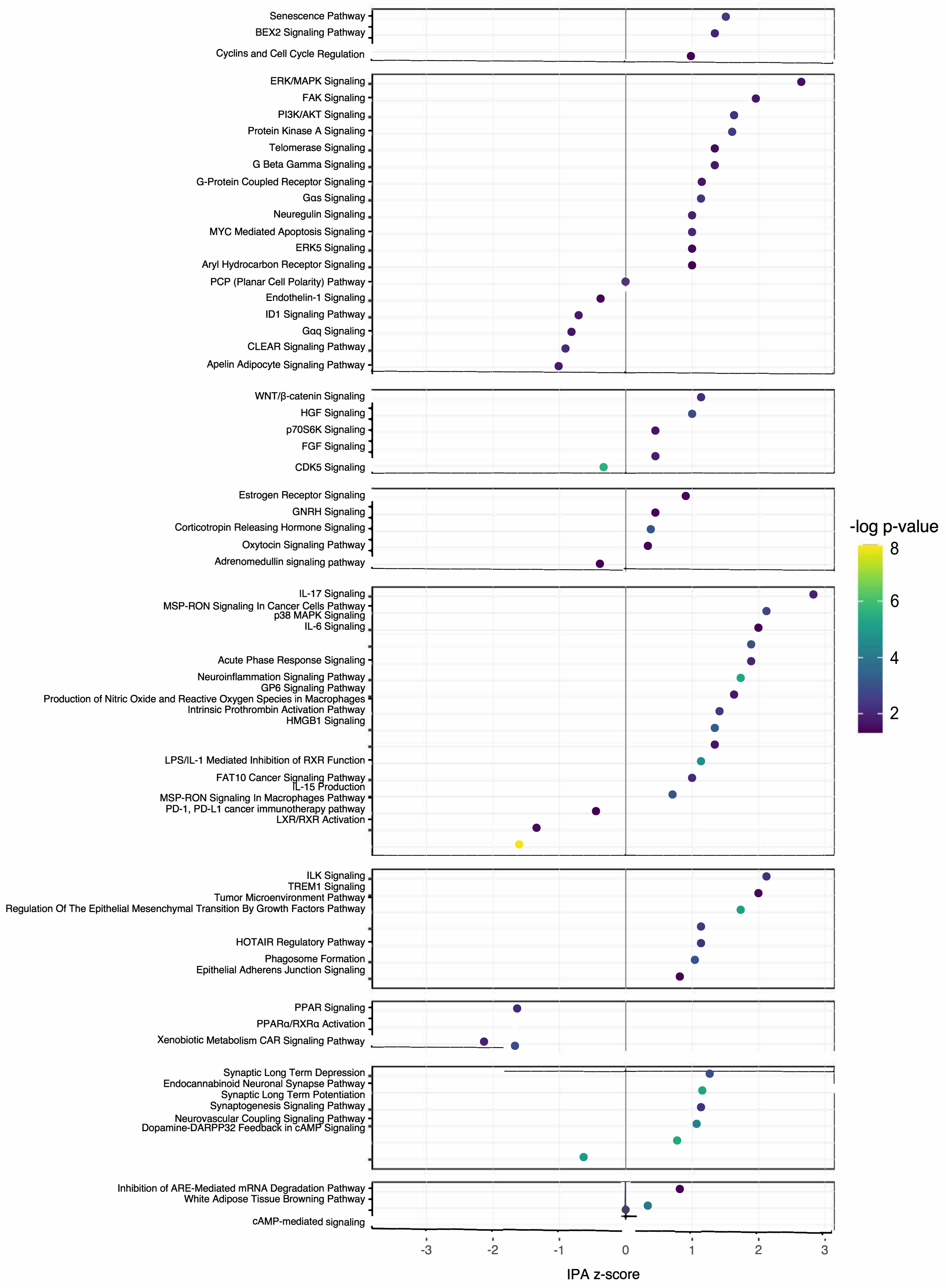

Supplementary Figure 5. Summary of differential miRNA analysis between Type 2 versus Type 1 tumors. Pathway analysis results generated using Ingenuity Pathway Analysis (IPA) of differentially expressed mRNA targets of miRNAs identified by differential miRNA analysis. Negative and positive z-scores (x axis) represent predicted decreased and increased activation of a canonical pathway, respectively. The -log p-value generated by IPA represents results of a Fisher's Exact Test analysis where $p<0.05$ indicates a statistically significant association.

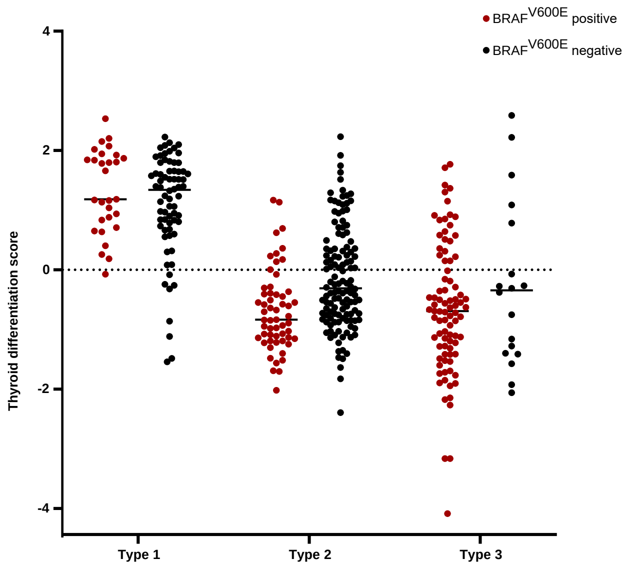

Supplementary Figure 6. Analysis of radioactive iodine in relation to BRAF mutation, molecular subtypes, and survival. Swarm plot for thyroid differentiation score in tumors with ($BRAF^{V600E}$+) and without ($BRAFV^{600E}$-) BRAF mutation within each molecular subtype.

Supplementary Tables

***Table S1**. List of 82 prognostic genes identified by the HighLifeR algorithm.*

Ensembl Id	Gene symbol	Cox coefficient	Median Wald
ENSG00000256340	ABCC6P1	0.520768309	7.29
ENSG00000006071	ABCC8	0.527365643	7.435
ENSG00000087008	ACOX3	-0.135262062	5.73
ENSG00000106351	AGFG2	-0.576857988	5.115
ENSG00000174939	ASPHD1	-0.056422136	7.89
ENSG00000143153	ATP1B1	-0.634637309	5.71
ENSG00000176171	BNIP3	0.481171543	6.365
ENSG00000169679	BUB1	0.486144916	6.51
ENSG00000174456	C12orf76	-0.398425798	7.5
ENSG00000140534	TICRR	0.48193524	5.215
ENSG00000005436	GCFC2	0.483266113	6.165
ENSG00000187699	C2orf88	0.39672355	5.49
ENSG00000102547	CAB39L	0.599622045	6.87
ENSG00000145386	CCNA2	0.451659864	6.415
ENSG00000134690	CDCA8	0.435330273	5.745
ENSG00000120334	CENPL	0.52238087	5.555
ENSG00000143375	CGN	0.116300399	5.57
ENSG00000159259	CHAF1B	-0.277546754	6.92
ENSG00000157224	CLDN12	0.493680008	8.05
ENSG00000166200	COPS2	0.563494199	7.195
ENSG00000109861	CTSC	0.550982366	5.47
ENSG00000157349	DDX19B	-0.114777881	6.57
ENSG00000154309	DISP1	-0.735991696	5.72
ENSG00000105877	DNAH11	0.194929732	7.96
ENSG00000101210	EEF1A2	0.449221618	6.09
ENSG00000144895	EIF2A	0.467681074	4.82
ENSG00000134899	ERCC5	0.542985514	7.585
ENSG00000010030	ETV7	-0.770455963	6.3
ENSG00000171824	EXOSC10	-0.316327768	5.82
ENSG00000106462	EZH2	0.134691239	7.94
ENSG00000198673	TAFA2	0.431047745	5
ENSG00000151876	FBXO4	0.515171874	5.25
ENSG00000158483	FAM86C1P	0.536269858	6.26
ENSG00000115414	FN1	0.328845266	6.61
ENSG00000157259	GATAD1	0.482462525	5.53
ENSG00000087258	GNAO1	-0.265909951	5.57
ENSG00000168243	GNG4	0.419481666	6.245
ENSG00000121957	GPSM2	0.867992205	6.33
ENSG00000178075	GRAMD1C	-0.205682846	6.17
ENSG00000172432	GTPBP2	0.084532581	8.7
ENSG00000163607	GTPBP8	0.483783136	5.295
ENSG00000203814	HIST2H2BF	0.273528922	7.74
ENSG00000197837	HIST4H4	0.465857517	5.595
ENSG00000123485	HJURP	0.406545042	5.385
ENSG00000100441	KHNYN	0.152422246	8.34
ENSG00000126775	ATG14	0.653695484	9.04
ENSG00000213213	CCDC183	0.167191052	5.14
ENSG00000090889	KIF4A	0.423793103	5.925
ENSG00000147592	LACTB2	0.224682185	6.14
ENSG00000132434	LANCL2	-0.155360547	9.45

ENSG00000215154	LOC652276	-0.045677263	5.89
ENSG00000188002	LOC728613	0.508392175	5.335
ENSG00000005189	REXO5	0.40011219	7.79
ENSG00000062524	LTK	0.525551983	7.265
ENSG00000155363	MOV10	-0.40299367	6.92
ENSG00000204410	MSH5	0.279058457	6.3
ENSG00000163719	MTMR14	0.184785974	6.19
ENSG00000204544	MUC21	0.121429373	7.41
ENSG00000095777	MYO3A	0.539377062	7.795
ENSG00000117650	NEK2	0.418364442	4.86
ENSG00000176953	NFATC2IP	0.484971897	9.07
ENSG00000136159	NUDT15	-0.153311689	9.85
ENSG00000132182	NUP210	0.590926993	5.74
ENSG00000177614	PGBD5	0.573618168	7.3
ENSG00000174236	REP15	-0.715414739	4.7
ENSG00000007384	RHBDF1	-0.274976868	7.02
ENSG00000177519	RPRM	0.396172279	5.285
ENSG00000155876	RRAGA	-0.442612866	4.825
ENSG00000271699	SNX29P2	0.463704934	5.71
ENSG00000109618	SEPSECS	0.532154942	5.95
ENSG00000165480	SKA3	0.404286547	5.425
ENSG00000149150	SLC43A1	0.55071094	7.49
ENSG00000164828	SUN1	0.326655982	6.26
ENSG00000138111	MFSD13A	-0.195472081	5.39
ENSG00000112742	TTK	0.401763376	5.02
ENSG00000140830	TXNL4B	0.449185245	6.06
ENSG00000124602	UNC5CL	0.249151891	5.61
ENSG00000071127	WDR1	-0.572758029	4.95
ENSG00000047644	WWC3	-0.531730013	5.45
ENSG00000158545	ZC3H18	0.246219417	5.53
ENSG00000149054	ZNF215	-0.60794384	8.6
ENSG00000177842	ZNF620	0.473448521	5.695

Table S2. *Univariate and multivariable analysis of factors associated with 5-year PFS in the validation set.*

Risk Factor	Univariable Analysis		Multivariable Analysis	
	Hazard Ratio (95 % CI)	*P*	Hazard Ratio (95 % CI)	*P*
Sex (female v. male)	1.37 (0.63 – 2.94)	0.43		
Age (< 50 years v. ≥50 years)	1.37 (0.66 – 2.86)	0.40		
Tumor size (≤4 cm v. >4 cm)	1.39 (0.64 – 3.01)	0.40		
Histological type				
Classical	1 (reference)			
Follicular	0.47 (0.14 – 1.58)	0.23		
Tall Cell	0.92 (0.28 – 3.05)	0.89		
Other	0.00 (0.00 – inf)	1.0		
T stage				
T1	1 (reference)		1 (reference)	
T2	3.15 (0.64 – 15.61)	0.16	4.33 (0.86 – 21.86)	0.08
T3	5.16 (1.19 – 22.34)	**0.03**	4.49 (0.98 – 20.58)	0.053
T4	11.80 (2.15 – 64.71)	**0.004**	3.27 (0.43 – 24.97)	0.25
N stage (N0/NX v. N1)	1.85 (0.86 – 3.98)	0.12		
M stage (M0 v. M1)	162.50 (10.16 – 2598)	**3.19×10^{-4}**	135.68 (6.49 – 2836.73)	**1.55×10^{-3}**
$BRAF^{V600E}$ mutation (Absent v. Present)	1.18 (0.57 – 2.45)	0.66		
TERT promoter mutation (Absent v. Present)	2.05 (0.71 – 5.90)	0.18		
Thyroid Differentiation Score				
< -1	1 (reference)			
-1 to 0	0.44 (0.15 – 1.31)	0.14		
> 0	0.33 (0.10 – 1.08)	0.07		
$BRAF^{V600E}$-RAS class				
BRAF-like	1 (reference)			
RAS-like	0.56 (0.16 – 1.93)	0.36		
ATA Risk				
Low	1 (reference)		1 (reference)	

Intermediate	2.19 (0.91 – 5.26)	0.08	1.31 (0.47 – 3.60)	0.60
High	3.82 (1.55 – 9.41)	**3.62×10^{-3}**	3.12 (0.91 – 10.79)	0.07
Molecular Subtype				
Type 2	1 (reference)		1 (reference)	
Type 1	0.69 (0.19 – 2.52)	0.58	0.93 (0.24 – 3.62)	0.92
Type 3	2.75 (1.25 – 6.08)	**0.01**	3.64 (1.55 – 8.56)	**3.06×10^{-3}**

HR = Hazard ratio, the ratio of recurrence rates in two comparator groups for any independent variable (for binary variables) or the logarithm of the change in death rate per unit change of the independent variable (if the variable is continuous).

CI = Confidence interval

Significant values are in bold ($p < 0.05$)

Table S3. Clinical characteristics of molecular subtypes assigned to the external validation dataset from Korea.

**Factor**	**Molecular subtype**			***P***
	**Type 1 (*N* = 41)**	**Type 2 (*N* = 69)**	**Type 3 (*N* = 14)**	
**Sex,** N (%)				0.98[a]
Male	11 (26.8)	18 (26.1)	4 (28.6)	
Female	30 (73.2)	51 (73.9)	10 (71.4)	
**Age,** mean ± SD	48.5 ± 13.3	47.5 ± 13.2	40.9 ± 9.7	0.15[b]
**Histological type,** N (%)				< 0.0001*[a]
Classical	12 (29.3)	50 (72.5)	14 (100.0)	
Follicular	29 (70.7)	19 (27.5)	0 (0.0)	
Tall Cell	0 (0.0)	0 (0.0)	0 (0.0)	
Other	0 (0.0)	0 (0.0)	0 (0.0)	
**Focality,** N (%)				0.22[a]
Unifocal	33 (80.5)	49 (71.0)	8 (57.1)	
Multifocal	8 (19.5)	20 (29.0)	6 (42.9)	
**Extrathyroidal spread,** N (%)				0.01*[a]
None	31 (75.6)	28 (40.6)	7 (50.0)	
Minimal	7 (17.1)	29 (42.0)	5 (35.7)	
Gross	3 (7.3)	12 (17.4)	2 (14.3)	
**T Stage,** N (%)				0.26[a]
T1	21 (51.2)	25 (36.2)	7 (50.0)	
T2	5 (12.2)	3 (4.3)	0 (0.0)	
T3	15 (36.6)	41 (59.4)	7 (50.0)	
T4	0 (0.0)	0 (0.0)	0 (0.0)	
**N Stage,** N (%)				0.11[a]
N0/NX	34 (82.9)	47 (68.1)	8 (57.1)	
N1	7 (17.1)	22 (31.9)	6 (42.9)	
**M Stage,** N (%)				0.81[a]
M0	40 (97.6)	67 (97.1)	14 (100.0)	
M1	1 (2.4)	2 (2.9)	0 (0.0)	
**RAS mutation,** N (%)				0.002*[a]
Absent	21 (60.0)	56 (84.8)	12 (100.0)	
Present	14 (40.0)	10 (15.2)	0 (0.0)	
**$BRAF^{V600E}$ mutation,** N (%)				0.01*[a]
Absent	21 (60.0)	23 (34.8)	2 (16.7)	
Present	14 (40.0)	43 (65.2)	10 (83.3)	
**$BRAF^{V600E}$-RAS class,** N (%)				< 0.0001*[a]
BRAF-like	13 (38.2)	54 (80.6)	14 (100.0)	
RAS-like	21 (61.8)	13 (19.4)	0 (0.0)	
**ATA Risk,** N (%)				0.009*[a]
Low	28 (68.3)	24 (34.8)	6 (42.9)	
Intermediate	11 (26.8)	37 (53.6)	8 (57.1)	
High	2 (4.9)	8 (11.6)	0 (0.0)	
**AMES,** N (%)				0.64[a]

Low	10 (24.4)	13 (18.8)	4 (28.6)	
High	31 (75.6)	56 (81.2)	10 (71.4)	
**MACIS score,** N (%)				0.59[a]
< 6.00	32 (78.0)	58 (84.1)	14 (100.0)	
6.00 to 6.99	6 (14.6)	9 (13.0)	0 (0.0)	
7.00 to 7.99	2 (4.9)	1 (1.4)	0 (0.0)	
> 8.00	1 (2.4)	1 (1.4)	0 (0.0)	

a = Pearson's Chi-squared test
b = Kruskal–Wallis test
* = significant

Table S4. Five-year recurrence rates for different cohorts.

Cohort	Molecular subtype		
	Type 1	Type 2	Type 3
**TCGA Discovery**			
Early	3/54 (5.6%)	1/73 (1.4%)	5/34 (14.7%)
Advanced	5/28 (17.9%)	1/82 (1.2%)	7/52 (13.5%)
All	8/82 (9.8%)	2/155 (1.3%)	12/86 (14.0%)
**TCGA Test**			
Early	1/22 (4.5%)	1/28 (3.6%)	4/12 (33.3%)
Advanced	2/15 (13.3%)	8/49 (16.3%)	12/37 (32.4%)
All	3/37 (8.1%)	9/77 (11.7%)	16/49 (32.7%)
**Korea cohort**			
Early	0/32 (0%)	0/46 (0%)	0/8 (0%)
Advanced	0/9 (0%)	1/23 (4.3%)	0/6 (0%)
All	0/41 (0%)	1/69 (1.4%)	0/14 (0%)
**Edmonton cohort**			
Early	0/11 (0%)	0/18 (0%)	2/12 (16.7%)
Advanced	0/11 (0%)	13/51 (25.5%)	5/29 (17.2%)
All	0/22 (0%)	13/69 (18.8%)	7/41 (17.1%)
**All cohorts combined**			
Early	4/119 (3.4%)	2/165 (1.2%)	11/66 (16.7%)
Advanced	7/63 (11.1%)	23/205 (11.2%)	24/124 (19.4%)
All	11/182 (6.0%)	25/370 (6.8%)	35/190 (18.4%)
